# Bacterial Ammonia Causes Significant Plant Growth Inhibition

**DOI:** 10.1371/journal.pone.0063538

**Published:** 2013-05-15

**Authors:** Teresa Weise, Marco Kai, Birgit Piechulla

**Affiliations:** University of Rostock, Institute of Biological Sciences, Rostock, Germany; Friedrich-Alexander-University Erlangen-Nurenberg, Germany

## Abstract

Many and complex plant-bacteria inter-relationships are found in the rhizosphere, since plants release a variety of photosynthetic exudates from their roots and rhizobacteria produce multifaceted specialized compounds including rich mixtures of volatiles, e.g., the bouquet of *Serratia odorifera* 4Rx13 is composed of up to 100 volatile organic and inorganic compounds. Here we show that when growing on peptone-rich nutrient medium *S. odorifera* 4Rx13 and six other rhizobacteria emit high levels of ammonia, which during co-cultivation in compartmented Petri dishes caused alkalization of the neighboring plant medium and subsequently reduced the growth of *A. thaliana*. It is argued that in nature high-protein resource degradations (carcasses, whey, manure and compost) are also accompanied by bacterial ammonia emission which alters the pH of the rhizosphere and thereby influences organismal diversity and plant-microbe interactions. Consequently, bacterial ammonia emission may be more relevant for plant colonization and growth development than previously thought.

## Introduction

The rhizosphere defines the area of soil directly bordering the plant roots. This is a preferred habitat for many microorganisms, which has been known to contain up to 10^11^ microbial cells per gram root [1]. A complex exchange of organic and inorganic molecules is a prerequisite for such strong microbial growth. Plants, for example, secrete 20–50% of photosynthetically assimilated carbon as root exudates in the form of sugars, flavonoids, aliphatic acids, amino acids, organic acids and proteins [2], [3], [4], [5]. Microorganisms metabolize these rhizosphere deposits and release products themselves that influence organisms of this habitat in many different ways [6], [7], [8]. In the last decade, several reports established that rhizobacteria also emit complex mixtures of volatiles; which in turn influence the development of plants, fungi and other organisms positively or negatively [9], [10], [11], [12], [13], [14], [15], [16], [17], [18], [19]. Subsequent analysis of the headspace volatiles of the bacteria revealed spectra of different composition and complexity [8]. Although in many cases the bioactive components have yet to be determined, four bacterial volatiles - 2,3-butanediol, acetoin, 2-pentylfuran and CO_2_ - were demonstrated to act as plant-growth-promoting compounds [9], [20], [21]. In contrast, dimethyl disulfide, 2-phenylethanol and hydrogen cyanide were reliably shown to act as phytotoxic volatiles [17], [19], [22], [23]. Hydrogen cyanide is released from many *Pseudomonas* spp. and *Chromobacterium* spp. [19], [24], and dimethyl disulfide and 2-phenylethanol were found in the volatile blends of many bacterial species [8].

A survey showed that particularly rich volatile mixtures were released from species of the genera *Chromobacter, Streptomyces* and *Serratia* [8]. The emission spectrum of *Serratia odorifera* 4Rx13, which was isolated from the rhizosphere of *Brassica napus* [25], comprises approximately one hundred volatiles [22]. Although many compounds could be detected, only a few were unequivocally identified, such as sodorifen, 2-phenylethanol, dimethyl disulfide, dimethyl trisulfide, methanethiol, methanol, ethanol and CO_2_. Among these, dimethyl disulfide and 2-phenylethanol were shown to reduce plant growth [17], [22]. However, respective growth reductions were only visible in Petri dish experiments when high doses of both compounds (e.g. IC_50_: 20 µg) were applied. Such DMDS and 2-phenylethanol levels, however, were not reached by bacteria growing on NB medium in the Petri dish. Therefore it was hypothesized that additional inhibiting volatiles were released by *S. odorifera* 4Rx13 and preliminary investigations suggested ammonia to play a key role [22]. Since the bacterial emission of ammonia was not intensively studied, yet, we surveyed nine bacterial species and delved into a possible contribution of ammonia influencing the growth of *A. thaliana*.

## Materials and Methods

### Organisms and the Co-cultivation of Bacteria and Plants

Bacteria which originated from the rhizosphere of potato or oilseed rape were selected: *Serratia odorifera* 4Rx13, *S. plymuthica* HRO-C48, *S. plymuthica* 3Re4-18, *Pseudomonas fluorescens* L13-6-12, *P. fluorescens* 3Re2-7, *Bacillus subtilis* B2g, *Stenotrophomonas maltophilia* R3089, *S. rhizophila* P69, *Staphylococcus epidermidis* 2P3-18a [11].

**Figure 1:**
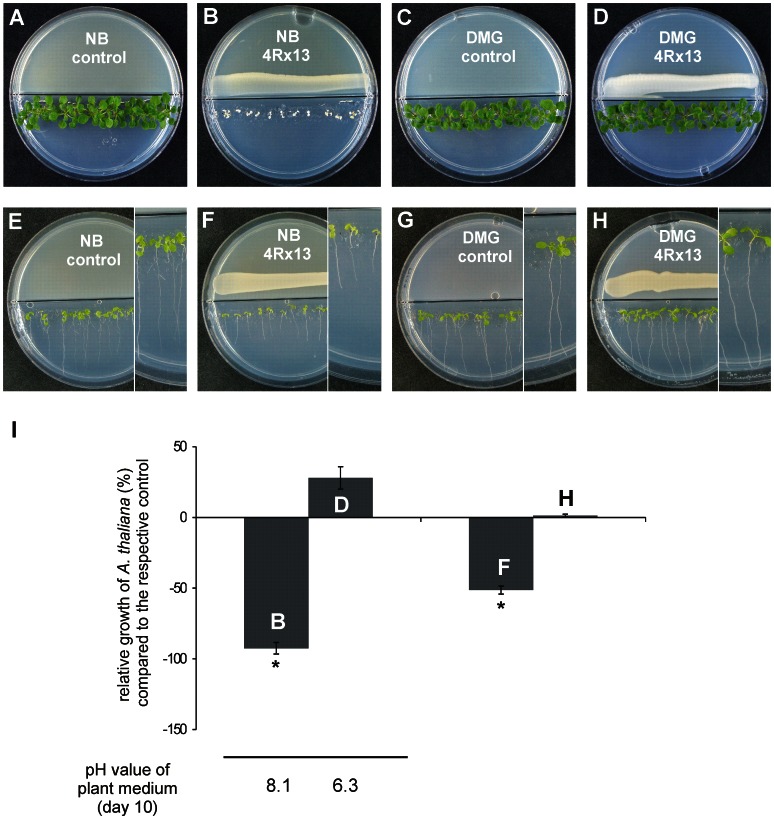
Growth of Arabidopsis thaliana Col-0 co-cultivated with Serratia odorifera 4Rx13. (a–d) Determination of shoot fresh weight of *A. thaliana* co-cultivated with *S. odorifera* 4Rx13. *A. thaliana* seedlings were placed on MS medium and *S. odorifera* 4Rx13 was applied near the plastic barrier on NB II (b) or DMG (d). (e–h) Determination of root fresh weight of A. thaliana co-cultivated with S. odorifera 4Rx13. Petri dishes were incubated vertically to allow better exploration of root growth. (a, e) and (c, g) were inoculated without bacteria. (i) Quantitative determination of the growth of *A. thaliana* after 10 days of co-cultivation. Relative increase/decrease of fresh weights and root lengths was calculated in comparison to plants that were not co-cultivated with bacteria (a, c, e, g = controls). Lower panel indicates the pH of the medium at the end of the experiment. Arithmetic means and standard deviations were calculated based on three experiments with five replicates. Significance (*) was calculated using Students t-test (p≤0.01). NB II: nutrient broth II; DMG: Davis-Mingioli+glucose = minimal medium with 55 mM glucose; MS: half strength of Murashige-Skoog plant medium.

Bacterial strains were cultivated either on nutrient broth (NBII) [11] or on synthetic medium (DMG) [26]. *Arabidopsis thaliana* Col-0 was sterilized and cultivated on Murashige-Skoog (MS) medium as described [12], [17], [22], [27]. In one experimental set up the plant medium was adjusted to pH 5, 6, 7 or 8 using NaOH (Fig. S2). Ten strains of *A. thaliana* Col-0 and 50 µl *S. odorifera* 4Rx13 (10^7^ cell ml^−1^) were co-cultivated in bipartite Petri dishes as described by Wenke and colleagues [17] (Fig. 1 and S1). To evaluate the influence of nutrients, NBII was supplemented with 10 mM, 50 mM and 100 mM glucose (Carl Roth, Karlsruhe, Germany; Fig. S1). For root analysis, bipartite Petri dishes were positioned vertically in the growth chamber to allow plant roots to grow without restriction. Plant growth was determined according to i) the primary root length after 5 days and ii) the fresh weight of shoots after 10 days of co-cultivation. The results were compared to control plants that were grown without the co-cultivation of bacteria.

### Determination of pH Values in the Agar and NH_3_ Emission of Different Bacteria

**Figure 2:**
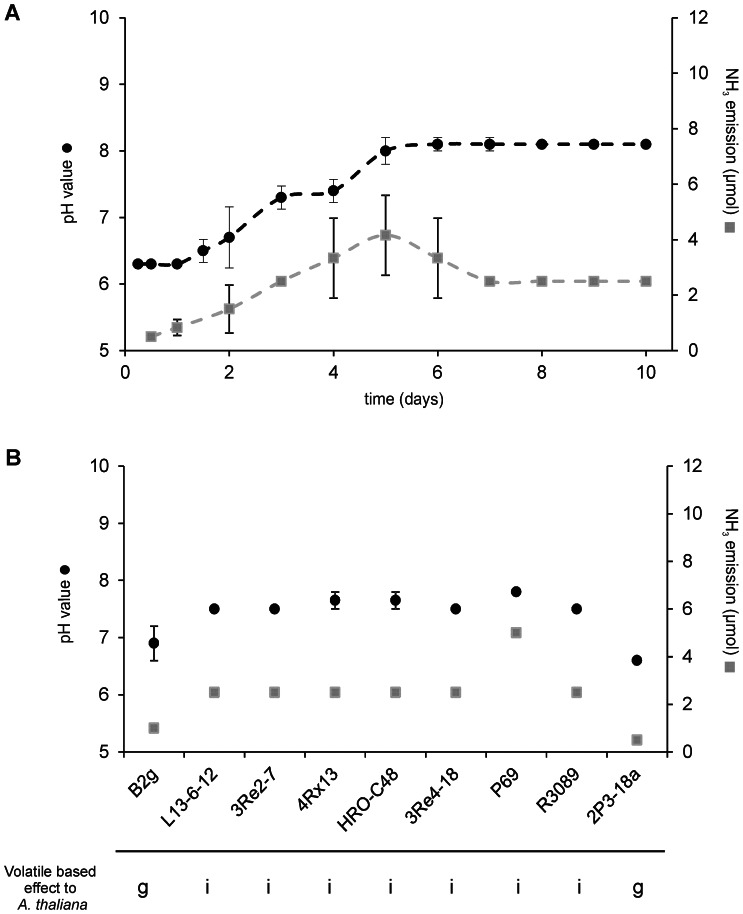
Emission of NH_3_ by Serratia odorifera 4Rx13 and pH shift of plant MS medium. (a) *S. odorifera* 4Rx13 was applied on NB II medium along the plastic barrier of the bipartite Petri dish. At indicated time points, ammonia (gray, dashed line) was quantified in the headspace of the second compartment using Quantofix test paper. The color changes were documented and compared with calibrated standard curve of 0.5 µmol, 1 µmol, 2.5 µmol, 5 µmol, 10 µmol and 50 µmol ammonia solutions [22]. The pH value of the plant MS medium in the second compartment was also determined (black, dashed line). NH_3_ emissions and pH values were determined during a time course of 10 days. Arithmetic means and standard deviations were calculated based on three experiments each with two replicates. NB II: nutrient broth II; MS: half strength of Murashige-Skoog plant medium. (b) NH_3_ emissions (gray square) and pH values in the MS medium (black square) were determined after 72 hours of growth of the following rhizobacteria: B2g - Bacillus subtilis, L13-6-12 - Pseudomonas fluorescens, 3Re2-7– Pseudomonas trivialis, 4Rx13 - Serratia odorifera, HRO-C48– Serratia plymuthica, 3Re4-18– Serratia plymuthica, P69– Stenotrophomonas rhizophila, R3089– Stenotrophomonas maltophilia, 2P3-18a - Staphylococcus epidermidis. Lower panel indicates A. thaliana inhibition (i) or growth (g) during co-cultivation with respective bacterial isolates.

The pH values of the media were determined by placing pH paper on the agar (Carl Roth, Karlsruhe, Germany) at different time points during cultivation (Fig. 2a, b). The ammonia emission was determined using Quantofix® ammonium test sticks (Macherey & Nagel, Düren, Germany) as described [22]. 50 µl of a bacterial culture (10^7^ cell ml^−1^) was applied as a line on NBII agar in one compartment of bipartite Petri dishes. After 72 hours of cultivation, a slit was cut into the wall of the empty compartment and the ammonium test stick was deposited opposite to the bacterial culture. The slit was sealed with Nescofilm® (Carl Roth, Karlsruhe, Germany) to avoid any loss of volatiles and contaminations. After two hours, a microliter syringe was inserted through the slit and Nessler reaction was initiated with 10 µl dH_2_O. After 30 sec, the chemical reaction was stopped by adding 10 µl of NaOH (32%). The color changes were documented and compared with calibrated standards of 0.5 µmol, 1 µmol, 2.5 µmol, 5 µmol, 10 µmol and 50 µmol ammonia solutions (Carl Roth, Karlsruhe, Germany) [22]. The NH_3_ emission of *S. odorifera* 4Rx13 was analyzed after 3 h, 6 h, 12 h, 24 h, and 48 h, and every 24 h after until 240 h (Fig. 2a). The NH_3_ production of nine rhizobacteria was determined after 72 h (Fig. 2b). To check whether NH_3_ might be responsible for pH-value changes of the agar and negative growth effects on *A. thaliana,* plants were cultivated with concentrations exchanged on a daily basis (see calibration standard) of ammonia solution (Fig. S3).

### Co-cultivation of Plants and Bacteria in the Presence of Phosphoric Acid (H_3_PO_4_)

**Figure 3:**
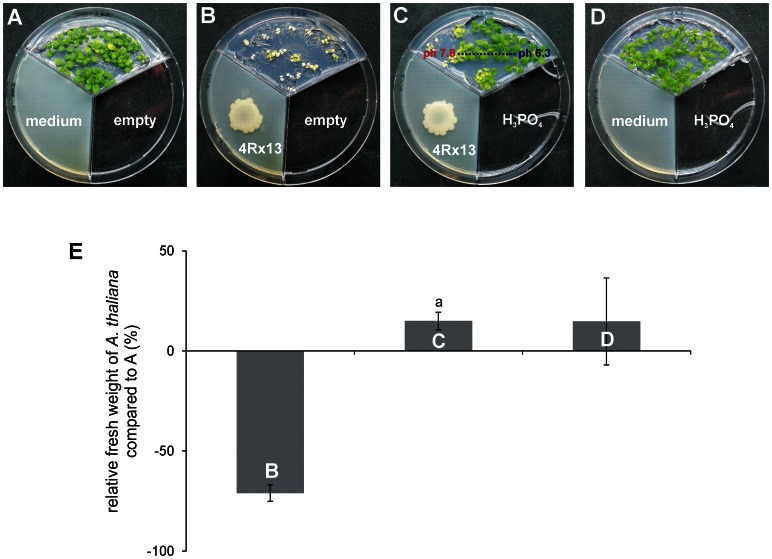
Co-cultivation of Arabidopsis thaliana Col-0 and Serratia odorifera 4Rx13 in the presence of phosphoric acid. Growth of *A. thaliana* cultivated on MS medium in tripartite Petri dishes for 10 days: (a) cultivation without bacteria and without phosphoric acid (control 1); (b) co-cultivation with bacteria and without phosphoric acid; (c) co-cultivation with bacteria and with phosphoric acid; (d) cultivation without bacteria and with phosphoric acid (control 2). (e) Quantitative determination of the growth of A. thaliana. Relative increase/decrease of fresh weights of shoots was calculated in comparison to shoots cultivated without bacteria and without phosphoric acid (a). Arithmetic means and standard deviations were calculated based on three experiments each with five replicates. (a) indicates significances of p≤0.05 (Student’s t-test) in comparison to the co-cultivation of plants and bacteria without phosphoric acid. NB II: nutrient broth II; MS: half strength of Murashige-Skoog plant medium.

To further confirm the hypothesis, we conducted co-cultivation experiments with phosphoric acid. Phosphoric acid reacts with ammonia to form ammonium phosphate salts [28]. 15 surface-sterilized and stratified *A. thaliana* seedlings were cultivated on MS agar for 72 h in the first compartment of tripartite Petri dishes (Fig. 3). In the second compartment, 20 µl bacterial culture of *S. odorifera* 4Rx13 (10^7^ cell ml^−1^) was spotted and the third compartment was filled with 5 ml 0.74 mM H_3_PO_4_ (Carl Roth, Karlsruhe, Germany). The fresh weight of shoots was documented after 10 days and compared with that of controls (Fig. 3a).

### Statistics

Arithmetic means were calculated based on three repeated experiments, each performed with two to ten replicates. Significances were evaluated using Student’s *t*-test. P-values are indicated in the figure captions.

## Results

It was previously documented that growth of *Arabidopsis thaliana* is inhibited by bacterial volatiles [12], [17], [23]. Chlorosis as well as the suppressed leaf and root development of *A. thaliana* were observed when the plants were co-cultivated with *S. odorifera* 4Rx13 on complex medium (NB II) (Fig. 1b and f, respectively), but no growth reduction was detected when the bacteria were cultivated on minimal medium supplemented with glucose (DMG) (Fig. 1d and h). This ‘glucose effect’ was corroborated by adding 10 to 100 mM glucose to NB II medium (Fig. S1); whereas strong inhibitory effects were obvious at low glucose concentrations, as glucose concentrations increased, growth retardation became less severe. It was concluded that *S. odorifera* 4Rx13 produced either different quantities or qualities of inhibitory or growth-promoting volatiles while growing on different media. In fact, the headspace volatiles of NBII were more complex and the profile was dominated by sodorifen [29], while only sparse amounts of sodorifen were present in the headspace of DMG (data not shown). Although contributing ca. 45% to *Serratia’s* blend, no plant growth alterations could be attributed to sodorifen, since the application of 0.2 µmol of pure sodorifen did not influence the growth of *A. thaliana* [22]. Interestingly, the growth inhibition of *A. thaliana* turned out to be only partially due to dimethyl disulfide and 2-phenylethanol [22], [17], and therefore it was concluded that other compounds of the bacterial blend must have inhibitory capabilities as well.

Routine determination of the pH of the plant medium gave the first hint of the identification of such a compound. Surprisingly, the pH of the plant medium increased from 6.3 to 8.1 upon volatile-mediated co-cultivation with *S. odorifera* 4Rx13 (Fig. S1g, lower panel). Furthermore, a correlation between plant growth inhibition and the alkalization of the plant medium was noticed. The plants grew well on media with pH of 5 and 6, while chlorotic phenotypes appeared when the plants were grown on media with elevated pH (Fig. S2). Consequently, it was hypothesized that bacterial volatiles may indirectly inhibit plant growth via the alkalization of the plant medium.

Ammonia was suspected to be responsible for this alkalization. In a time course experiment, bacterial ammonia and amine emissions were determined using Nessler’s reaction (Fig. 2a). A strong positive correlation between ammonia emission and pH alteration of the plant medium was observed between day 1 and day 5, while between day 5 and 10, ammonia emission slightly decreased and the pH of the medium remained at elevated levels. This decrease of ammonia emission is most likely due to growth cessation due to nutrient limitations. To further verify that the ammonia emanation of *S. odorifera* 4Rx13 was responsible for a pH shift of the plant medium, ammonia was scavenged by phosphoric acid to form ammonium phosphate salts [28]. Indeed, when *A. thaliana* was co-cultured with *S. odorifera* 4Rx13, and phosphoric acid was applied into the third compartment, the growth of the plant was restored (Fig. 3). Furthermore, the application of 1 µmol or higher amounts of commercially available ammonia to the test system retarded plant growth significantly (Fig. S3). The chlorotic phenotype, the decrease of fresh weight and the increased pH values of the plant medium in combination with the neutralization of these effects by ammonia removal indicate that ammonia caused plant growth inhibition indirectly via the alkalization of the plant medium.

In light of these results ammonia emissions of nine bacteria and the subsequent pH shifts in the plant media were determined (*S. odorifera* 4Rx13, *S. plymuthica* HRO-C48, *S. plymuthica* 3Re4-18, *Pseudomonas fluorescens* L13-6-12, *P. trivialis* 3Re2-7, *Stenotrophomonas rhizophila* P69 and *Stenotrophomonas maltophilia* R3089, *Staphylococcus epidermidis* 2P3-18a and *Bacillus subtilis* B2g). All tested bacterial strains except *B. subtilis* and *S. epidermidis* emitted ammonia at substantial levels; the emission by *S. rhizophila* P69 was especially pronounced (Fig. 2b). The high ammonia release generated a pH shift in the plant medium, which correlated with the negative growth effects of *A. thaliana*, while the low ammonia emission of *B. subtilis* B2g and *S. epidermidis* 2P3-18a resulted in small or no pH shifts and had no effect on plant growth. These results substantiated the observation that bacteria growing on peptone-rich media released ammonia in concentrations that were sufficient to alkalize the MS medium which in turn retarded plant growth.

## Discussion

This paper demonstrates i) the potential of ammonia emission by rhizobacteria and ii) its consequences for the growth and development of *Arabidopsis thaliana* in volatile-mediated co-cultivations with bacteria.

Ammonia can be produced by nitrite ammonification [30], by the degradation of various amino acids utilized from proteins of food or of complex media [31], by the decarboxylation of amino acids to produce biogenic amines as well as ammonia [32], by deamination, and by the urease-mediated hydrolytic degradation of urea [33]. The genome of *S. odorifera* 4Rx13 encodes more than 55 putative ammonia-producing enzymes, including ammonia lyases, amino acid and nucleotide deaminases, nitrilases, nitrite reductases, pyridoxamine phosphate oxidases, and amino acid deaminases, which strongly support the process of ammonia synthesis. Similar enzyme activities are also expected to be present in the other eight bacterial species investigated here.

Ammonia fulfills several biological roles. In addition to its important metabolic role in many organisms, ammonia’s toxicity is well known. Ammonia seems not to be toxic for non-phototrophic bacteria even at rather high levels (over 100 mM); in contrast cyanobacteria and plants tolerate only low levels [34]. One prerequisite for toxic functionality appears to be its rapid diffusion through the majority of biological membranes [33]. Due to the rather lipophilic character of the uncharged NH_3_ molecule, the rapid permeation is biologically significant even at small concentration differences across the membrane. When ammonia accumulates in the plant cells at levels higher than 0.1 mM, plants showed symptoms such as the chlorosis of leaves, a lowered root/shoot ratio, stimulated root branching, declined mycorrhizal associations, and inhibited seed germination and seedling establishment [35].

The mechanisms that underlie these phenotypic aberrations are manifold; among them are biochemical pH-stat systems that account for differences in the internal H^+^ balance and decreasing external pH due to NH_4_^+^ acquisition. In contrast to the acidification of the rhizosphere, the co-cultivation experiments presented here demonstrated significant plant damage due to the alkalization of the medium (ca. pH 8) as a consequence of bacterial ammonia emission. It was previously shown that NH_3_ entered plant cells very rapidly in the presence of a high external pH, inducing transient elevations of cytoplasmic and vacuolar pH [36], [37]. Furthermore, NH_3_ treatment at high pH levels stimulated the increase of cytoplasmic calcium concentrations due to Ca^2+^ influx through the plasma membrane or Ca^2+^ release from internal stores; subsequently altering the calcium homeostasis [38]. It seems very likely that due to the continuous production and emission of NH_3_ into the headspace, similar events occurred in *A. thaliana* during its volatile-mediated co-cultivation with *S. odorifera* 4Rx13.

The volatile-based bacterial-plant interactions are, however, more complex and cannot only be explained by the emission of ammonia. *Stenotrophomonas rhizophila* P69 for example, emits much more ammonia compared to *S. odorifera* 4Rx13 (Fig. 2b), but the reaction of *A. thaliana* to the volatiles of both bacteria were almost the same [12], suggesting that other volatiles of *S. rhizophila* may compensate the effects of ammonia. Another discrepancy was detected: *Bacillus subtilis* B2g emits approximately 1 µmol of ammonia at the third day which shifted the plant medium to pH 7 (Fig. 2b) and the daily application of 1 µmol ammonia in a bipartite Petri dish resulted in 95% plant growth reduction (Fig. S3), while according to Vespermann and colleagues [12] the growth of *A. thaliana* was not affected by the volatiles of *B. subtilis* B2g at day 10. These apparent inconsistencies may partially be explained by the different experimental set ups, e.g. i) time points of examination were different (3 days vs. 10 days), ii) daily ammonia applications are different to continuous production of volatiles by the bacteria and iii) the application of a single volatile ammonia most likely generates different reactions in the plant compared to a complex volatile mixture emitted by bacteria.

In addition to the above mentioned indirect action mode, bacterial ammonia may also operate directly on the roots and/or leaves. Uptake must be facilitated by ammonium transporters. Six members of an AMT family are known in *A. thaliana*: five genes are expressed in roots and one gene is expressed in pollen [39], [40], [41]. Each of these transporters has its own affinity profile to ammonia, suggesting specific physiological functions [42]. A working model hypothesized that the external ammonium signal is conferred to the cytosolic side, via either a membrane-anchored receptor-like kinase or a transceptor (a protein that acts as a transporter and receptor at the same time). Consequently, the root length of *A. thaliana* was reduced during volatile-mediated co-cultivation with bacteria (Fig. 1) [17], [23], which correlated well with observations of stunted roots due to NH_4_^+^ contact with the primary root tip; such contact arrested root growth by inhibiting cell elongation rather than cell division [43].

Organismal diversity and plant-microbe interactions may depend on bacterial ammonia emission. Experiments have to be conducted which monitor bacterial derived ammonia in the rhizosphere to elucidate ammonia depending processes in the subterranean zone of plants.

## Supporting Information

Figure S1 **Growth of** ***Arabidopsis thaliana*** **Col-0 volatile-mediated co-cultivated with** ***Serratia odorifera*** **4Rx13 growing on different glucose concentrations.** *A. thaliana* was co-cultivated with S. odorifera 4Rx13. *A. thaliana* seedlings were placed on MS medium and *S. odorifera* 4Rx13 was applied near the plastic barrier on NB II (b–e) or DMG (f). NB II was supplemented with glucose at indicated concentrations (c–e). Fresh weights of the shoots were determined after 10 days of co-cultivation. (g) Quantitative determination of the growth of *A. thaliana*. Relative increase/decrease of fresh weights of shoots was calculated in comparison to shoots which were cultivated without bacteria (a). Arithmetic means and standard deviations were calculated based on three experiments each with five replicates. A–f indicate significances of at least p≤0.05 (Student’s t-test) in comparison (a) to respective control plants, (b) to NB II, (c) to NB II +10 mM glucose, (d) to NB II +50 mM glucose, (e) to NB II +100 mM glucose and (f) to DMG. Lower panel: pH values of MS medium after 10 days of co-cultivation (b–f). pH values were analyzed by placing pH indicator paper on agar. The pH of the MS medium in the control experiment (a) remained at 6.3 throughout the experiment. NB II: nutrient broth II; DMG: Davis-Mingioli+glucose = minimal medium with 55 mM glucose; MS: half strength of Murashige-Skoog plant medium.
(TIF)

Figure S2 **Growth of** ***Arabidopsis thaliana*** **Col-0 on MS medium of different pH.** (a–e) *A. thaliana* was cultivated on MS medium of pH = 5, pH = 6, pH = 7, pH = 8 and pH = 9 for 10 days. (f) Quantitative determination of growth of *A. thaliana*. Relative fresh weights of shoots were calculated in comparison to the weight of plants grown at pH 6 (b = 100%). Arithmetic means and standard deviations were calculated based on three experiments each with seven replicates. Significances were calculated using Student’s *t*-test in comparison to b (a: p≤0.001; b: p≤0.005). MS: half strength of Murashige-Skoog plant medium.
(TIF)

Figure S3 **Growth of** ***Arabidopsis thaliana*** **Col-0 in the presence of NH_3._** (a–g) Growth of ten surface-sterilized and stratified *A. thaliana* in the presence of different amounts of ammonia (0 to 50 µmol NH_3_ solved in 10 ml water). *A. thaliana* seedlings were placed on MS medium and ammonia was filled into the upper compartment of a bipartite Petri dish. Every 24 h the solution was exchanged and freshly prepared ammonia solution was applied. (h) Quantitative determination of the growth of *A. thaliana* after 10 days of co-cultivation. Relative increase/decrease of fresh weights of shoots was calculated in comparison to the weight of shoots cultivated without ammonia (a). Arithmetic means and standard deviations were calculated based on three experiments each with five replicates. Significances (*) were calculated using Student’s *t*-test in comparison to control (a) and 0.5 µmol NH_3_ (b), p>0.01. Lower panel: pH values of the MS medium 10 days after cultivation (b–g). pH values were determined by placing pH indicator paper on agar. The pH of the MS medium in the control experiment (a) remained at 6.3 throughout the experiment. MS: half strength of Murashige–Skoog plant medium.
(TIF)
